# After Harm: A Plea for Moral Repair after Algorithms Have Failed

**DOI:** 10.1007/s11948-025-00555-y

**Published:** 2025-09-18

**Authors:** Pak-Hang Wong, Gernot Rieder

**Affiliations:** 1 Academy of Chinese, History, Philosophy and Religion, Faculty of Arts and Social Sciences, Hong Kong Baptist University, Kowloon Tong, Hong Kong; 2 Centre for the Study of the Sciences and the Humanities, University of Bergen, Bergen, Norway

**Keywords:** Algorithmic harm, Algorithmic imprint, AI ethics, AI governance, Moral repair, Post-harm scenarios

## Abstract

In response to growing concerns over the societal impacts of AI and algorithmic decision-making, current scholarly and legal efforts have mainly focused on identifying risks and implementing safeguards against harmful consequences, with regulations seeking to ensure that systems are secure, trustworthy, and ethical. This preventative approach, however, rests on the assumption that algorithmic harm can essentially be avoided by specifying rules and requirements that protect against potential dangers. Consequently, comparatively little attention has been given to post-harm scenarios, i.e. cases and situations where individuals have already been harmed by an algorithmic system. We contend that this inattention to the aftermath of harm constitutes a major blind spot in AI ethics and governance and propose the notion of algorithmic imprint as a sensitizing concept for understanding both the nature and potential longer-term effects of algorithmic harm. Arguing that neither the decommissioning of harmful systems nor the reversal of damaging decisions is sufficient to fully address these effects, we suggest that a more comprehensive response to algorithmic harm requires engaging in discussions on moral repair, offering directions on what such a plea for moral repair ultimately entails.

## The Problem

In the past couple of years, AI-driven algorithmic decision-making has colonized ever more areas of human life. Driven by a relentless pursuit of efficiency and optimization, the *automation of society* (see AlgorithmWatch, 2020) has progressed at a rapid pace, with modern data analytics *transforming how we live*,* work*,* and think* (see Mayer-Schönberger & Cukier, 2013). This transformation, however, is not without its challenges and, after much debate and sensitization in both the media (see, e.g., Angwin et al., 2016) and academia (see, e.g., Barocas & Selbst, 2016), there is now widespread recognition that algorithmic decision-making can undermine fundamental rights and subject individuals to considerable *harm*.

To date, scholarly and legal efforts have primarily focused on *anticipating*, *identifying*, and *preventing* the adverse impacts of algorithmic systems, with regulations seeking to ensure that such systems are “secure, trustworthy, and ethical” (EU, 2024, p. 2). This *preventative approach*, however, rests on the assumption that algorithmic harm can essentially be avoided by specifying rules and requirements that safeguard against potential risks and negative impacts. Consequently, comparatively little attention has been given to *post-harm scenarios*, that is, cases and situations where individuals have already been harmed by an algorithmic system. While major ethics guidelines and governance recommendations do mention the need for accountability through feedback, traceability, and redress mechanisms (see, e.g., AI HLEG, 2019; UNESCO, 2022), the absence of any real detail reduces the perceived significance of such requirements to an afterthought in texts that are firmly committed to a “safety-first mindset” (NIST, 2023, p. 18) and almost exclusively centre on precautionary measures. The problem with this relative inattention to the *aftermath of harm* is twofold.

First, as demonstrated by numerous real-world cases, harm through algorithmic processes is not an abstract concern, but a well-documented, well-researched, and increasingly pervasive threat in today’s data-driven society (see McGregor, 2021; Shelby et al., 2023). By anticipating certain kinds of harm, we can work towards mitigating potential risks, but no matter how well one prepares and how effective the safeguards are, risk can never be fully eliminated. Given this, to eschew serious engagement with post-harm obligations in favour of precautionary policies seems both conceptually and empirically flawed. Rather, it would be prudent to consider that, as Perrow (1984) famously argues, when systems are tightly coupled and complex – features that both apply to modern algorithmic systems (see Bianchi et al., 2023) – it is unavoidable, even “normal,” that things go wrong. If we further take into account that algorithmic harm is often not accidental but the accepted by-product of purpose-built systems (see, e.g., Zweig, 2022 , p. 155ff) or the result of malintent (see, e.g., Blauth et al., 2022), it becomes evident that safety can never be complete and that attending to the aftermath of harm is not a case of too little, too late, but an acknowledgment that there is indeed a need to *set things right* if prevention failed.

Second, the brevity with which response measures are presented and discussed neither adequately reflects the potential severity of algorithmic harms nor the nature of these harms nor the difficulties involved in addressing them. While the report-trace-redress protocol is positioned as a seemingly straightforward solution, it ultimately raises more questions than it answers. After all, are algorithmic damages really always so obvious and clear-cut that they can be easily articulated? Given the high complexity and dynamic nature of advanced algorithmic systems, should technical audits still be necessary for establishing responsibility? And what does “redress” actually entail? At a minimum, then, there is need for further discussion and clarification of these requirements. Arguably, however, the problem with the specified requirements does not only lie in their brevity and vagueness but also in the underlying rationale. In essence, it appears that the envisaged response measures are distinctly conducive to a streamlined operational logic where mistakes are reported, corrected, and, if there is liability, compensated (cf. Weinberg, 2022). Yet this assumes that algorithmic harms can generally be managed in such an optimized manner, which, as we shall demonstrate below, is not always the case. Quite to the contrary, a closer look at cases of algorithmic harm would suggest that damages can be much more severe and impactful than current ethics and governance frameworks recognize. What is thus needed is a broader but also a more thoughtful approach to governing algorithmic harm, an approach that is more sensitive and responsive to the actual nature and consequences of such harms and that engages in deeper discussions on how these harms can be adequately addressed.

In this short provocation, we want to present the notion of algorithmic imprint as a sensitizing concept for understanding both the nature and potential longer-term effects of algorithmic harm. We then proceed to argue that neither the decommissioning of harmful systems nor the reversal of damaging decisions is sufficient to fully address these effects. Finally, we suggest that a more comprehensive response to algorithmic harm requires engaging in discussions on moral repair, offering directions on what processes of moral repair should ultimately encompass.

## Tracing Algorithmic Harm: On the Impact of Algorithmic Imprints

In a recent paper, Ehsan et al. (2022) introduce the notion of the *algorithmic imprint* to illustrate how merely removing or modifying a harm-inflicting algorithmic system does not necessarily undo or mitigate its consequences. Rather, the detrimental effects may “extend well beyond the algorithm’s ‘lifetime’,” leaving their mark on the “mental wellbeing of data subjects long after stopping [its] use” (ibid., p. 1). They exemplify this point through an in-depth case study of the Ofqual grading controversy in Bangladesh, where even though the much-criticized algorithmically-normed grades were eventually globally rescinded by the UK exam boards (see Weale & Stewart, 2020), the affected students still felt a “bitter aftertaste” and “deep sense of injustice” even after their grades improved (Ehsan et al., 2022, p. 8). Thus, while students appreciated the revisions after having experienced downgrading-by-algorithm, they “could not ignore what the process had put them through” (ibid.). Teachers expressed similar sentiments as they, rather than the UK boards responsible for the grading system, had been blamed and villainized by the students, feeling like a “punching bag, taking the blow for something [they] had no voice in” (ibid., p. 7). After the algorithm was scrapped and the grades were revised, many students apologized to their teachers in an effort to rectify the damaged teacher-student-relationship, yet teachers remained resentful towards the boards for using them “as human shields” (ibid., p. 8).

What is so instructive about the study of the Ofqual case and the notion of the algorithmic imprint is that they provide a sense of the lingering effects of algorithmic harm as well as a vocabulary to articulate and address them. Importantly, while Ehsan and co-authors’ discussion pays particular attention to the algorithm’s afterlife – that is, the period after deployment ceases – they emphasize that such imprints “develop and evolve throughout deployment” (ibid, p. 2), their contours forming while a system is still in use. Thus, an imprint-aware approach does not necessarily imply a conceptual refocusing from operational to decommissioned systems, but merely highlights that problems can persist even when algorithmic deployments have stopped, asking us to think of algorithmic harm as an enduring wound or injury rather than a temporally discrete, one-off occurrence. As the authors write, the “death of the algorithm does not entail the death of the issues it created.” (ibid.: 9) Even when an algorithm is removed, its “ghost” may still be there, impacting people’s well-being and how they perceive and continue to engage with algorithmic operations.

In addition to *reframing* and *broadening* how we assess algorithmic impacts, Ehsan and co-authors suggest that imprint-awareness can inform algorithm design and governance by serving as a guiding tool and sensitizing developers, operators, regulators, and end-users to the potential real-world remnants of algorithmic creations. However, while it seems crucial to exercise caution and foresight in an effort to avoid harmful designs and lower the likelihood of damaging events, such proactive measures yet again do not account for what ought to happen once the damage is done. Put differently, even though the authors astutely point to the destructive traces that algorithmic systems may leave on people’s lives, their recommendations remain largely within the preventative logic of current AI policies, positioning an “imprint-aware design mindset” as a means to make the “development process more human-centered and sociotechnically-informed” (ibid.: 9). Having previously argued for value sensitive design approaches as a means to detect and prevent algorithmic biases (see Simon et al., 2020), we concur with this perspective but think that the example of the Ofqual algorithmic grading controversy offers a chance to also reflect more deeply on what actions to take when harm has occurred and its consequences endure.

​If we take the lessons from the Ofqual controversy and the idea of algorithmic imprints seriously, it becomes obvious that the currently prevailing responses to cases of algorithmic harm – that is, the decommissioning of tools or systems, the reversal of decisions, and the compensation of (groups of) individuals – are not necessarily sufficient to deal with the lingering effects of such harms. How so?

On the one hand, the purpose of decommissioning a harmful algorithmic system is to ensure that no further damage is done by stopping the system from interacting with and acting upon individuals (see Tartaro, 2023; Johnson et al., 2024). Decommissioning, however, neither resolves the existing damage nor does it account for any negative consequences that may persist beyond the system’s termination. Furthermore, by reinforcing the assumption that harm ceases once the system shuts down, an overly strong focus on decommissioning as a sufficient response risks sidelining discussions on the prolonged effects of algorithmic harm and the need for proper accountability. This of course is not to suggest that decommissioning does not play a crucial role in addressing and responding to instances of algorithmic harm, but rather to point out that decommissioning alone is not enough and cannot serve as the sole response.

The reversal of algorithmic decisions, on the other hand, seeks to return an individual’s well-being to its state before any negative effects occurred. When the damage caused by such decisions is substantial and mere reversal cannot fully remedy the individual’s condition, compensation payments may serve as a supplementary measure to rectify the situation (see Gardner, 2022). While appealing on paper and potentially well-intentioned, the notion that harm can be easily erased or offset rests on an overly simplistic, transactional understanding of harm that neither fully captures nor sufficiently accounts for the emotional and social dimensions of the situation. As the Ofqual controversy clearly demonstrates, even after the reversal of the algorithmically-normed grades, both students and teachers continued to express discontent, frustration, and a sense of injustice. As Ehsan and co-authors stress in their discussion, there simply “is no easy ‘undo’ button for algorithmic deployments” (Ehsan et al., 2022, p. 9), and while monetary compensation can be an important remedial measure, it should not be seen as a complete solution.

In contrast to prevailing approaches, we contend that a deeper consideration of algorithmic harm and its consequences demands a *reorientation from a transactional to a moral and relational perspective*, focusing not just on balancing losses but on actively restoring what has been broken. In particular in cases of serious wrong involving an unjust and morally indefensible treatment of individuals (see Diberardino et al., 2024), redistributive action will be insufficient to deal with moral and relational harm because such harm calls for a restoration of the injured party’s moral standing and requires rebuilding a just and equal relationship between the wrongdoers and the wronged (see Cohen, 2020; Fornaroli, 2024). In other words, addressing algorithmic wrong requires those responsible to reinstate the rights of the affected individuals and reaffirm their moral status as equal members of the community. Consequently, tackling the after-effects of algorithmic harm must extend beyond the decommissioning of systems, the reversal of decisions, and the provision of financial compensation, incorporating approaches and strategies for *moral repair*.

## A Plea for Moral Repair after Algorithms Have Failed

In her seminal work *Moral Repair*, Walker (2006) offers a detailed discussion of the basic elements that sustain moral relationships, focusing on the need to rebuild trust and hope after wrongdoing has occurred and moral bonds have been fractured.^1^ Importantly, she starts from the assumption that “no wrong is ever undone,” and that it is only “the sequel to the wrong that either ‘does right’ by the victim or does not do so” (ibid., p. 7). Consequently, repairing moral bonds “cannot mean return to the status quo,” but must “aim at bringing morally diminished or shattered relations to morally adequate form” (ibid., p. 26f). Moving from a situation of loss and damage to the restoration of moral ties, however, requires more than fixing mistakes and providing restitution, necessitating a wider range of actions aimed at affirming and reassuring victims of harm. More specifically, Walker identifies six tasks for moral repair: (1) placing responsibility on wrongdoers and others who share responsibility for wrongs; (2) acknowledging and addressing the wrong and harm caused to victims and communities; (3) (re)instating moral terms and standards within communities where wrong has caused doubt about the authority of those standards; (4) replenishing or creating trust among individuals in the recognition of shared moral standards and in their responsiveness to those standards; (5) igniting or nourishing hope that moral understandings and those who share them are worthy of trust; and (6), to the extent possible, (re)connecting in adequate moral relationships those who have done wrong and those who have been harmed (see ibid., p. 28).

From this list of tasks, it is apparent that moral repair is not just about revising errors and offering reparations, but about nurturing and protecting a background of trust and hope on which moral relations depend. Beyond formal accountability measures, this may require *rituals of recognition and respect* that provide voice, validation, and vindication to the victims of harm (see ibid., p. 18f; p. 35). For example, listening to victims’ stories, practices of public truth-telling, the extension of official apologies, show of sincere repentance, and guarantees of non-repetition can play as crucial a role in reweaving a broken relationship’s moral fabric as material compensations. Embedding such gestures into cultural practice may also help counter the perception that financial restitution alone is sufficient to resolve moral transgressions, pushing beyond a purely transactional view of restorative justice. The above list of tasks also reveals that moral repair ultimately requires both individual and communal efforts. While wrongdoers must accept responsibility for their actions, commit to reparative measures, and work toward rebuilding the damaged trust relationship, communities must equally provide acknowledgment and vindication by recognizing the harm, ensuring accountability, and lending credence to the expectation that the authority of shared norms and standards will be upheld. In particular in cases where wrongdoers refuse to take responsibility, it will be up to the community to reestablish moral order, imparting a sense of hopefulness that injustices will not go unaddressed. As Walker puts it, such hopefulness is not “the icing on the cake,” but “the pan in which the cake is baked.” (ibid., p. 210) When trust is crushed – such as through remorseless wrongdoing – there remains hope that others may yet be worthy of trust. When hope is crushed, however, a moral world cannot be sustained. (see ibid., p. 27) 

We contend that the *moral repair of algorithmic harm* ought to approximate the above list of tasks. More specifically, in the context of algorithmic harm, moral repair:

1. involves identifying and holding to account those responsible for harm, which may include developers, deployers, and those who have authorized the use of the algorithmic system;
2. requires wrongdoers and those who share responsibility to acknowledge and address the harm done to individuals, which may include not only decommissioning, reversal of decisions, and compensation, but also a range of moral gestures such as (public) admissions and apologies, a commitment to making amends and seeking reconciliation, and assurances of non-recurrence, all aimed at both validating and relieving the potential suffering, anger, and alienation of victims of wrong;
3. considers the state of moral norms and standards within a community, their absence or insufficiency when a system causes harm, and the need to reaffirm their relevance and value to those affected as well as to society at large;
4. focuses on restoring confidence among individuals that shared moral standards will be respected in the design and implementation of algorithmic systems and that efforts to encourage and enforce moral conduct will be supported;
5. calls for rebuilding reasonable hope that moral understandings and those responsible for upholding them are worthy of trust, providing assurance that principles of fairness and justice will be maintained;
6. aims to reconnect on both practical and moral grounds those who have done wrong and those who have been harmed, working toward a *climate of mutual respect* that in cases of serious injury may be difficult to establish.

Crucially, pursuing moral repair in cases of algorithmic harm should not be regarded as an optional or secondary concern. If those responsible for harm, those who share responsibility, and those tasked with upholding norms and values refuse or neglect to engage in at least attempting moral restoration, the consequences may be a gradual erosion of trust—not only in a specific algorithmic system and its providers—but also in the authorities that govern and regulate these systems, and in algorithmic practices as such.^2^ Given the recent rapid advancements in AI technology and the strong push for algorithmic services across both public and private sectors, such a loss of trust would seem especially concerning. Genuine efforts toward moral repair, however, could contribute to demonstrating trustworthiness even when safeguards have failed and harm occurs, counteracting processes of public alienation while providing a sense of justice for those who have been harmed.

When contemplating the repair of algorithmic harm, it is important to recognize the complex socio-technical nature of such harm and the challenges that ensue. In particular, advanced algorithmic systems are said to introduce a *responsibility gap* that makes it difficult to attribute responsibility, accountability, and/or culpability for their harmful consequences (see Matthias, 2004; Santoni de Sio & Mecacci, 2021). Since these systems operate with some degree of autonomy, determining who (or what) is responsible for the harm—whether it is the developers, the users, the (training) data, the systems themselves, or some combination of these parties and components—can be challenging. Moral repair faces the same challenge, given that one of its initial tasks involves identifying responsibility and acknowledging the harm done. In many cases, however, no specific individual or entity can be held directly responsible, as the harm arises from the interaction of multiple human and non-human actors (Shelby et al., 2023). While a satisfactory response to the responsibility gap remains a significant challenge to the field of AI ethics more broadly, we contend that efforts at moral repair ought not to be paralyzed by it, as we cannot simply blind ourselves to the lingering effects of algorithmic harm or dismiss them as inconsequential. Fortunately, Walker’s account of moral repair offers a constructive path forward by emphasizing the role of the community in upholding moral standards and restoring hope. Following Walker, we propose the notion of *communal responsibility* as a tentative response to the problem of the responsibility gap in the context of algorithmic harm: when individual responsibility is obscured or insufficient, the responsibility of moral repair falls to the community to validate and provide relief to the victims, to reaffirm shared moral norms and standards, and to ensure that injustice and unfairness do not go unaddressed (cf. Vallor & Ganesh, 2023).

Returning to the Ofqual case, it should now be clear that simply reversing the grades is insufficient for any serious attempt at moral repair. Additional measures could and should include a clear acceptance of responsibility, the issuing of (public) apologies to students and teachers, efforts to reaffirm the importance of fairness in grading and examination, and the provision of assurances that such an incident will not recur, all aimed at restoring confidence in the education system. In addition, developers, deployers, and others who share responsibility for the harm ought to engage directly with students and teachers, demonstrating their commitment to respecting their rights and to establishing a morally just and equitable relationship. Ultimately, failing to undertake these steps not only allows a broken – perhaps even shattered – moral relationship to persist but also actively compounds the harm, adding insult to injury. As Walker (2006, p. 19f) emphasizes,

[w]hen these responses are not forthcoming, the victim’s situation is worse than unaddressed, it is aggravated. If the community or authority to whom the victim looks for validation and vindication ignores the victim [or] treats the victim’s complaint as of little import, […] the victim will feel abandoned and isolated. That abandonment is a ‘second injury’ that can be humiliating.

## Concluding Remarks

In this provocation, we sought to draw attention to a significant oversight in prevailing discussions on the ethics and governance of algorithmic systems, namely the neglect of post-harm scenarios and the need for moral repair. Contrary to current understandings of algorithmic harm as temporally isolated occurrences, we aimed to demonstrate that such harms may have lingering effects and that the ‘afterlife’ of algorithmic systems can be as consequential as their initial and immediate impact. Following the work of Ehsan et al. (2022) and drawing on the Ofqual grading controversy, we thus argued for algorithmic imprint awareness, emphasizing that such awareness should not only inform the development and deployment of algorithmic systems – another precautionary measure – but spark reflection and conversation on how to adequately respond to the temporally extended consequences of algorithmic harm. We referred to this task, following Walker’s (2006) pioneering work on the topic, as one of moral repair, which represents a marked shift from the prevalent offender-centric report-trace-redress protocol to asking what victims of harm actually need from both the offender and society at large. Such a change in perspective also involves a reorientation from merely correcting mistakes and offsetting damages to actively seeking to restore moral bonds and public trust, reinforcing hope that whenever (algorithmic) harm does occur, it will at least be properly acknowledged and addressed. Unfortunately, the moral repair of algorithmic harm is not a straightforward matter as the complex sociotechnical nature of algorithmic systems with their inherent opacity and unpredictability will pose difficult theoretical and practical challenges to a satisfactory account of moral repair in cases of algorithmic harm. Nevertheless, we believe that this is a critical issue that researchers and policymakers *must* confront. It is only through such deeper reflection and engagement that we can start moving toward more realistic governance frameworks that recognize that risks can never be fully eliminated, dare to look beyond prevention, and commence taking responsibility for the full spectrum of algorithmic harm.

## Data Availability

No dataset was generated in completing the current paper.

## Footnotes

1 In this paper, we focus on Walker’s account, as we believe it offers the most comprehensive framework for understanding moral repair currently available. Other notable accounts of moral repair have been proposed for specific domains, e.g. Nancy Berlinger’s (2005) influential work on moral repair in the context of medical errors or Vives-Gabriel and co-authors’ (2023) recognition-based theory for corporate wrongdoing. In addition, recent philosophical analyses of apology also provide critical insights into specific facets of moral repair (see, e.g., Cohen, 2020). However, none of these works examine moral repair in relation to algorithmic harm, underscoring the need for the present discussion.

2 Here, we do not wish to suggest that individuals trust or distrust algorithmic systems per se. There are ongoing philosophical debates on whether trusting non-human artefacts is plausible (see Nickel et al., 2010; Tallant, 2019), as well as about the meaning of ‘trust’ and ‘trustworthiness’ in recent initiatives around ‘trustworthy AI’ (see, e.g., Reinhardt, 2023; Durán & Pozzi, 2025), which we cannot settle in this paper. Elsewhere, we have defended the view that trust in algorithmic systems should be understood as trust in *socio-technical systems* that are assemblages of human beings, technologies, and institutions (see Rieder et al., 2021; cf. Ananny, 2016). Consequently, when algorithmic harm is not sufficiently accounted for, the resulting loss of trust is rarely confined to a single component. Rather, it can lead to an erosion of trust more broadly, thereby undermining the communal foundations on which “a large part […] of everyday living rests” (Walker, 2006, p. 149).
